# Nature-Assembled Structures for Delivery of Bioactive Compounds and Their Potential in Functional Foods

**DOI:** 10.3389/fchem.2020.564021

**Published:** 2020-09-24

**Authors:** Alejandra Acevedo-Fani, Anant Dave, Harjinder Singh

**Affiliations:** ^1^Riddet Institute, Massey University, Palmerston North, New Zealand; ^2^International Iberian Nanotechnology Laboratory, Braga, Portugal

**Keywords:** casein micelle, milk fat globule, oleosome, bioactive compound, functional food

## Abstract

Consumers are demanding more natural, healthy, and high-quality products. The addition of health-promoting substances, such as bioactive compounds, to foods can boost their therapeutic effect. However, the incorporation of bioactive substances into food products involves several technological challenges. They may have low solubility in water or poor stability in the food environment and/or during digestion, resulting in a loss of their therapeutic properties. Over recent years, the encapsulation of bioactive compounds into laboratory-engineered colloidal structures has been successful in overcoming some of these hurdles. However, several nature-assembled colloidal structures could be employed for this purpose and may offer many advantages over laboratory-engineered colloidal structures. For example, the casein micelles and milk fat globules from milk and the oil bodies from seeds were designed by nature to deliver biological material or for storage purposes. These biological functional properties make them good candidates for the encapsulation of bioactive compounds to aid in their addition into foods. This review discusses the structure and biological function of different nature-assembled carriers, preparation/isolation methods, some of the advantages and challenges in their use as bioactive compound delivery systems, and their behavior during digestion.

## Introduction

Consumers are becoming increasingly conscious of their health and of planetary health. The alignment of a healthy diet with an eco-diet will be pivotal in the next decade. A rapidly growing segment of the population is considering different aspects when making their food choices; for example, no artificial ingredients and synthetic chemicals in their food products (the clean label trend) and products with eco-friendly attributes (efficient use of the materials, recycled/renewable content, and low energy and water waste), among others (Mintel, 2020). In this context, natural materials that can meet some of these requirements need to be identified and utilized by the food industry for new product development.

Bioactive compounds have received incredible attention within the scientific community and the food industry for their use as ingredients in food products. They are known to be both essential and non-essential compounds that occur in nature, are part of the food chain, and have been shown to have an effect on human health (Biesalski et al., 2009). Numerous biological functions have been attributed to these substances, including antioxidant, antithrombotic, anticoagulant, anti-inflammatory, antiproliferative, antihypertensive, antidiabetic, and cardioprotective activities, among others; these functions have been linked to the role of bioactive compounds in the prevention of chronic diseases, including cancer and cardiovascular diseases. Phytochemicals (carotenoids, polyphenols), micronutrients (vitamins A, D, E, and C, zinc, magnesium), fatty acids (omega-3, omega-6), and bioactive proteins (lactoferrin, lactoglobulin) are some examples of substances that have been recognized to have health-enhancing properties (Espín et al., 2007; Cencic and Chingwaru, 2010).

Functional foods are often used to refer to foods that can provide health-enhancing effects in addition to their basic nutrition. The health-enhancing properties come from bioactive compounds that are naturally present in the foods (e.g., anthocyanins in blueberries, hydroxytyrosol and its derivatives in olive oil, and docosahexaenoic acid and eicosapentaenoic acid in oily fish). The concentrations of these compounds in natural foods are generally low but may be further boosted by controlling their growing conditions (plant-origin) or through breeding techniques (animal-origin). However, the delivery of truly unique health benefits will probably require genetic engineering, which is not yet accepted by consumers. Although bioactive compounds are found naturally in many foods such as fruits, vegetables, seeds, whole grains, legumes, and marine products, it is not always practical to incorporate sufficient levels of these beneficial substances into the diet. Another approach is the addition of concentrates or extracts of the identified compounds to everyday products to enable the delivery of characterized and quantified levels into the final product, which can then provide a desired health-enhancing function.

The global functional food market is projected to grow to US$275.77 billion by 2025, with an increasing demand for nutritional and fortified foods being the major growth driver (Grand-View-Research-Inc., 2019). To meet the growing demand for functional foods, the food industry must address several critical challenges, including discovering the potential bioactivity of beneficial compounds, establishing optimal intake levels, and developing adequate food-delivering matrices and product formulations. The development of functional foods also faces many regulatory challenges.

In particular, the process of fortifying foods with extracted bioactive compounds changes the quality attributes and functionality of the product. Chemical stability of most bioactive compounds is greatly affected by pH, temperature, oxygen, light, or specific chemicals, which may lead to the loss of their biological functions (Loveday and Singh, 2011; Aizpurua-Olaizola et al., 2016; Singh, 2016). In addition, the absorption of bioactive substances is influenced by their solubility in the gastrointestinal tract, their stability during digestion, and their intestinal permeability (Gleeson et al., 2016). Therefore, many of these compounds may have poor bioavailability because of several physicochemical and physiological processes that occur after intake in the gastrointestinal tract (Rein et al., 2013). Within the food product, interactions between bioactive compounds and other food components are likely to occur during processing, storage, and transport. Such interactions may enhance or compromise the taste, color, and texture of enriched foods, and the release of bioactive compounds into the gastrointestinal fluids.

A strategy that is known to overcome some of these challenges is the entrapment of bioactive molecules within a delivery vehicle. The development of encapsulation technologies (micro- and nanoencapsulation) for improving the delivery of bioactive substances has been the subject of intensive research for many years (Han et al., 2011; Munin and Edwards-Lévy, 2011). These technologies seek to protect the bioactive compounds from environmental stresses, from interactions with the food matrix, and from gastrointestinal conditions that affect their bioactivity (Singh et al., 2009). Although some of these technologies have shown great success for some bioactive components, the fabrication method often involves laborious procedures, high energy consumption, or the use of non-natural materials (e.g., synthetic surfactants), which may cause rejection by consumers.

A different approach has recently been proposed; it uses nature-assembled structures for the delivery of bioactive compounds, in response to the need to find green, biocompatible, and natural materials. Nature-assembled colloidal structures are far more complex that those that have been engineered by colloidal scientists. These nature-origin structures are found in the animal and plant kingdoms; their purpose is to deliver the biological materials that are essential for physiological functions. They may offer several advantages as the encapsulation vehicles for bioactive compounds, compared with engineered delivery systems fabricated by encapsulation technologies. In this review, structures found in nature that have great potential for the encapsulation of bioactive compounds are discussed, in particular, those found in milk (casein micelles and milk fat globules) and in plants (oil bodies or oleosomes).

## Nature-Assembled Structures in Milk

Milk is a fluid that is secreted by the female of all mammalian species to supply the complete nutritional requirements of the neonate for its growth; milk is a rich source of lipids, lactose, proteins, minerals, vitamins, and water. Cows' milk comprises about 87% water and 13% total solids; the principal total solids are lactose (4.8%), fat (3.7%), and protein (3.4%). Fat and lactose provide the energy to the newborn whereas proteins provide a source of essential amino acids (Fox, 2003). The mineral phase is only a small fraction of milk (8–9 g/L), but is of major importance because minerals are essential for the growth and development of bones and teeth and play a major role in the structure and stability of milk proteins (Gaucheron, 2005; Lucey and Horne, 2009). The constituents of milk can be dissolved in the serum phase (e.g., lactose and most inorganic salts), or in a colloidal form (proteins), or as an emulsion (lipids) (Fox, 2009). The structures of proteins and lipids are designed by nature to deliver essential nutrients and bioactive compounds in a specific way to provide optimal health benefits. In the following sections, we discuss the structural features and the functionality of some of the colloidal structures present in milk and their potential application as delivery systems of bioactive molecules.

### Casein Micelles: A Natural Nanodelivery System

#### Biological Function, Composition, and Structural Features

The casein proteins in milk exist as assembled structures, called casein micelles, that are designed by nature to deliver essential nutrients, in particular, calcium and phosphate, to the neonate. The key biological functions of the casein micelle are considered to be the following (Holt et al., 2013):

- The safe secretion of high concentrations of calcium and phosphate in milk, so that the mammary gland does not become calcified.
- The secretion of high concentrations of potentially fibrillogenic casein proteins through the mammary gland.
- The coagulation of micelles in the stomach of the neonate, so that nutrients can be delivered for absorption at an optimal rate.
- The protection and transport of low-molecular-weight hydrophobic molecules (e.g., lipid soluble vitamins).

Four distinct types of casein, which are characterized by different polypeptide chains, are present in the casein micelles: α_S1_-, α_S2_-, β-, and κ-caseins. The structure, chemical properties, and functionality of the caseins have been extensively reviewed (Swaisgood, 2003; Horne, 2019; Rehan et al., 2019). All four caseins have a distinctly amphipathic character, with separate hydrophobic and hydrophilic domains, and have relatively open and unordered secondary structures. The lack of secondary and tertiary structure and their high flexibility have been attributed to the high level of proline residues in the amino acids composition of the caseins, i.e., 17, 10, 35, and 20 moles of proline per mole of α_S1_-, α_S2_-, β-, and κ-casein, respectively. All caseins (except κ-casein) are highly phosphorylated, with α_S1_-, α_S2_-, and β-caseins having 8–9, 11, and 5 phosphoserine residues per molecule, respectively. The phosphoserine residues are negatively charged, and are often found in clusters of two, three, or four residues, referred to as phosphoseryl clusters. These are the main sites for the binding of divalent cations, especially Ca^2+^.

Because of their unusual structural characteristics, along with their calcium-binding abilities, caseins assemble naturally into roughly spherical particles, known as casein micelles (Horne, 2009). The casein micelle comprises 94% protein, and the remaining 6% is mostly calcium and phosphate, with some magnesium and citrate, commonly referred to as micellar calcium phosphate (MCP). The micelles contain several thousand individual casein molecules, with their size ranging from 50 to 500 nm; their average size, as measured by electron microscopy, is around 150 nm, whereas dynamic light scattering measurements give an average diameter of around 200 nm (Anema and Klostermeyer, 1996; de Kruif and Holt, 2003). The mass of casein micelles ranges from 10^6^ to 10^9^ Da (average 10^8^ Da), their concentration in milk is 10^14^-10^16^ micelles/mL, and they are highly hydrated with 3.5 g H_2_O/g protein (Jeurnink and De Kruif, 1993). As this association of the casein proteins into casein micelles involves mainly electrostatic and hydrophobic interactions, changes in pH, temperature, and ionic strength induce changes in the balance between these two types of interaction, and can lead to changes in the casein micelle structure (De la Fuente, 1998). The casein micelles are stable to moderate heating or cooling treatments, but treatment with proteolytic enzymes or by acidification results in the formation of a curd or coagulum.

Various models for the structure of casein micelles have been debated over many years, giving different points of view (de Kruif and Holt, 2003; Farrell et al., 2006; Horne, 2006; Fox and Brodkorb, 2008; Dalgleish, 2011). One of the fundamental questions that needs to be addressed is how large numbers of casein molecules assemble in a controlled manner to give rise to stable particles that do not aggregate infinitely. One common and well-accepted feature of the different models is that the κ-casein molecules, which represent 15% of the total caseins, have to be located in such a way that they are able to stabilize the calcium-sensitive caseins (α_S1_-, α_S2_-, and β-caseins), consequently limiting the growth of the casein micelles. Another common feature concerns the role played by MCP in the casein micelle structure; because the removal of MCP causes the micelle to disintegrate into smaller particles, MCP must hold the casein molecules together. Many of the functional properties of the micelles depend on the properties of the surface, rather than on those of the interior, and, to some extent, the micelles can be regarded as hard spheres with a protective coating (de Kruif, 1999). In contrast, the interior of the micelle becomes important when we consider them as carriers of bioactive molecules, and, in this context, an understanding of the interior structure may be important.

**Figure 1:**
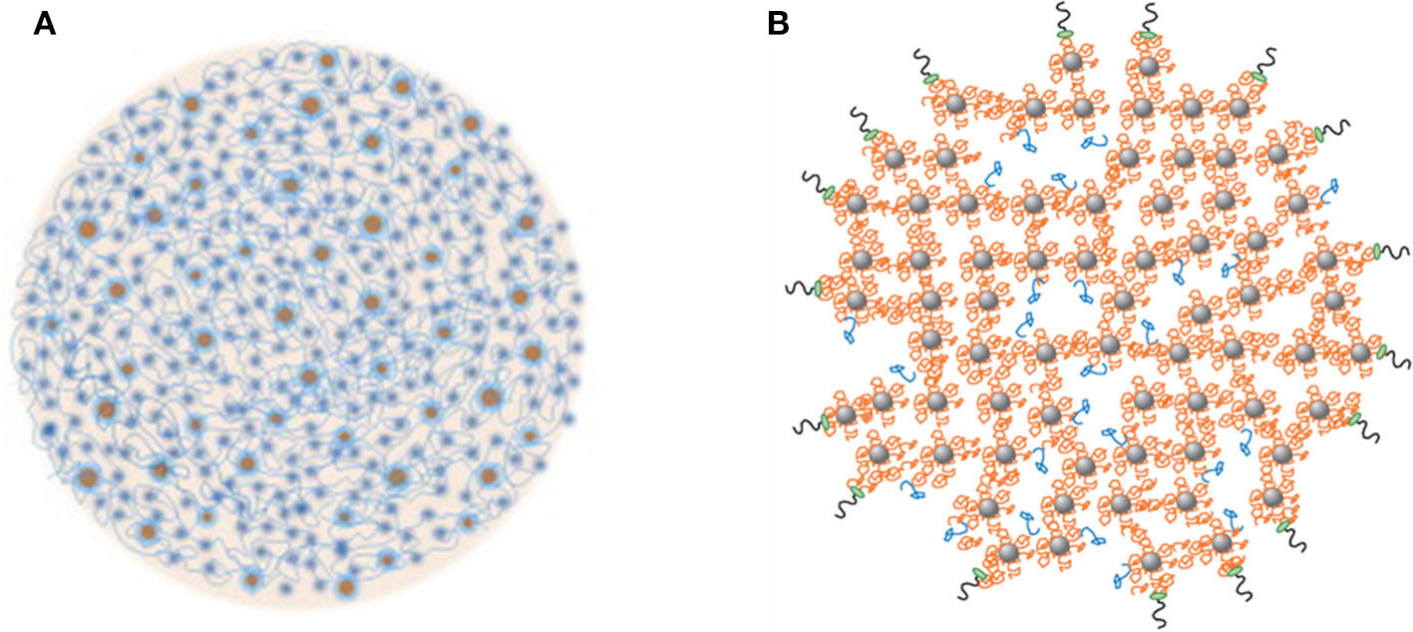
Two recent models of casein micelles. **(A)** the spheres represent the calcium phosphate nanoclusters, and the strands represent the casein network, reproduced with permission from de Kruif et al. (2012), copyright (2012), Elsevier Inc. **(B)** β- and α_S_-caseins (orange strands) are bound to the calcium phosphate nanoclusters (gray spheres), some β-casein are bound via hydrophobic interactions to other caseins (blue); the κ-casein molecules are bound on the surface of the casein micelles (black and green). Reproduced with permission from Dalgleish and Corredig (2012), copyright (2012), Annual Reviews Publishers.

Some casein micelle models have been proposed and discussed in published reviews (Dalgleish, 2011; Dalgleish and Corredig, 2012; de Kruif et al., 2012). Most of these are derived from the original nanocluster model proposed by Holt and Horne (1996). Two recent models are shown in Figure 1. The common features of these models are the presence of water channels, cavities, and high-density nanoclusters within the micelles. The casein micelles are porous structures that are made up of a matrix of casein molecules stabilized by calcium phosphate nanoclusters. The phosphorylated residues of the β- and α_S_-caseins are bound to the calcium phosphate nanoclusters, preventing uncontrolled growth and the precipitation of calcium phosphate, whereas the hydrophobic tails of the caseins can associate through hydrophobic interactions to form the protein network (Dalgleish, 2011). κ-Casein is believed to limit this self-association process and to stabilize the surface of the casein micelles. A debate remains on how many phosphoserine residues in a cluster are required to bind to the calcium phosphate nanoclusters and the answer actually determines how many linkage sites are present per casein molecule (Horne, 2009). A debate also remains on the width of the water channels and the size of the pores inside the micelles (de Kruif et al., 2012).

#### Disruption of Casein Micelles

The structure of the casein micelle is dynamic and responds to changes in the micellar environment, temperature, and pressure. Cooling the casein micelle to below 5°C causes significant solubilization of β-casein, and this process is reversible. Significant disruption of the micelles can be achieved by the extremes of acidic and alkaline pH treatments, because of changes in the solubility of calcium phosphate and electrostatic interactions (Huppertz et al., 2008). The addition of ethanol (>35%) at above 60°C in aqueous solution (O'Connell et al., 2001; Trejo and Harte, 2010) and the addition of chelating agents (e.g., EDTA or citrate) or urea also cause casein micelle dissociation. High pressure can significantly reduce the size of the casein micelles by disrupting the micellar structure with high energy input (Huppertz et al., 2004, 2006).

Some of these micelle dissociation–association properties could be manipulated to allow the binding/encapsulation of nutrients and bioactive molecules in the interior of the casein micelle structure. Moreover, the presence of several cavities (~20–30 nm in diameter) and channels (>5 nm in diameter) within the casein micelle structure (Trejo et al., 2011) offers new possibilities for the incorporation of bioactive molecules into the micelle interior. However, the highly porous nature of the casein micelles and the vulnerability of their structure to changes in the environmental conditions restrain their applications as delivery systems. A few attempts have been made to create more stable casein micelles, by cross-linking the caseins within the casein micelles with the enzyme transglutaminase or with a cross-linker, called genipin. Transglutaminase creates casein micelles (referred to as nanogel particles) consisting of a covalently linked casein network from which MCP can be removed without compromising its structural integrity (Silva et al., 2014). With genipin treatment, covalently linked casein particles can be formed, via cross-linking of the lysyl and arginyl residues of the casein molecules. These casein particles are smaller, are more negatively charged, and have smoother surfaces than natural casein micelles (Silva et al., 2014). These modified casein micelle structures offer interesting possibilities in applications in which integrity and biocompatibility may be important.

#### Casein Micelles as Carriers of Bioactive Compounds

Most of the studies to date concerning the entrapment/encapsulation of hydrophobic bioactive molecules by casein micelles have used the self-assembly approach, using sodium caseinate or isolated caseins as the starting material. Generally, a hydrophobic bioactive molecule, dissolved in an organic solvent, is mixed into a sodium caseinate solution. Specific amounts of C_6_H_5_K_3_O_7_, K_2_HPO_4_, and CaCl_2_ are then added to this solution to allow interactions of the caseins with these minerals and to create “artificial” casein-micelle-like particles. These so-called “reassembled casein micelles,” produced by various methods, have been shown to entrap hydrophobic molecules, such as triclosan, Vitamin D_2_, quercetin, β-carotene, sesamol, and docosahexaenoic acid (Semo et al., 2007; Roach et al., 2009; Zimet et al., 2011) (Table 1). It must be pointed out that these “reassembled casein micelles” may be structurally very different from the natural casein micelles synthesized in the mammary gland, and we recommend that they be referred to as casein aggregates or particles, not to be confused with the casein micelles.

**Table 1:** Some recent studies reported on casein micelles as delivery systems of hydrophobic compounds.

**Natural carrier**	**Bioactive compound**	**Encapsulation/binding method**	**References**
Native casein micelles	β-carotene	Injection of β-carotene/ethanol solution into casein dispersion, pH and temperature control	Moeller et al., 2017
	vitamin D_2_	Injection of vitamin D_2_/ethanol solution into casein dispersion and high-pressure treatment (0.1 or 600 MPa at 37 °C for 60 min)	Menéndez-Aguirre et al., 2011
Reassembled casein micelles	β-carotene	Injection of β-carotene/acetone into reassembled casein micelles	Sáiz-Abajo et al., 2013
	Sesamol	Noncovalent binding of sesamol to sodium caseinate and reassembly of casein micelles	Basurto et al., 2018
	vitamin D_2_	Noncovalent binding of ergocalciferol to sodium caseinate and reassembly of casein micelles	Semo et al., 2007
	Quercetin	Injection of quercetin/ethanol solution into reassembled casein micelles	Ghatak and Iyyaswami, 2019
	docosahexaenoic acid (DHA)	Non-covalent binding of DHA to sodium caseinate and reassembly of casein micelles	Zimet et al., 2011
	Triclosan	Triclosan/ethanol solution mixed with skim milk and treated by high pressure homogenization.	Roach et al., 2009
β-casein micelles	Curcumin	Curcumin/ethanol added to camel β-casein	Esmaili et al., 2011
	Naringenin	Ethanol injection	Li et al., 2019

Of the major casein proteins, β-casein is the most hydrophobic and has an amphiphilic nature, with a short polar head (N-terminal) and a hydrophobic tail (C-terminal). The polar N-terminal domain contains four of the five phosphoserine residues, grouped in one cluster of sequence Glu–SerP–Leu–SerP–SerP–SerP–Glu–Glu. This region is therefore negatively charged (net charge of −11.5 at pH 6.6) and hydrophilic. Because of this unique primary structure, β-casein has the tendency to self-associate into micelle-like structures, with the hydrophobic tail of the molecule buried inside the micelle and the hydrophilic head sticking out, creating an electrostatic repulsion layer (de Kruif and Grinberg, 2002; O'Connell et al., 2003). β-Casein micelles have been widely studied for the delivery of several hydrophobic compounds. In all of these studies, the solubility of the hydrophobic compounds was improved after the binding of these compounds within the hydrophobic core (Table 1). For example, vitamin D was bound into β-casein micelles directly, via hydrophobic interactions, and the binding strength varied with pH and ionic strength (Forrest et al., 2005). Folic acid interacted with β-casein by hydrophobic contacts, and its photodecomposition was reduced (Zhang et al., 2014). Naringenin was shown to interact spontaneously with β-casein via hydrophobic interactions and van der Waals' forces and hydrogen bonds (Moeiniafshari et al., 2015).

A few studies have explored the use of native casein micelles as carriers of nutrients and bioactive compounds. Sahu et al. (2008) separated the casein micelles from raw skim milk by ultracentrifugation and then used the casein micelle fraction as an encapsulating agent to deliver curcumin to cancer cells. The formation of the complex between casein micelles and curcumin was attributed to hydrophobic interactions. Mohan et al. (2013) showed that natural casein micelles have the ability to associate with hydrophobic vitamin A. Cheema et al. (2015) reported that native casein micelles isolated from raw milk exhibited a stronger binding affinity toward hydrophobic compounds, including sphingomyelins, phosphatidylcholines, and phosphatidylethanolamines. Clearly, a further understanding of the ability and the mechanism of the binding of hydrophobic molecules within casein micelles would allow us to create novel nanometer-scale structures that would be suitable for the delivery of bioactive compounds.

With respect to hydrophilic materials, casein micelles have a strong ability to bind metal ions and other charged molecules. One of the key micronutrients of interest is iron, as this is considered to be the most difficult micronutrient for food fortification. Iron has high reactivity with the protein and lipid components of food, often resulting in rancidity, lipid oxidation, and protein aggregation (Hurrell, 2002). A number of studies on the interactions of iron with caseins and casein micelles have been published (Usami et al., 2011; Mittal et al., 2016); some of these studies were aimed at developing the encapsulation and delivery of iron by complexing it with caseinate or the casein micelles in milk.

**Figure 2:**
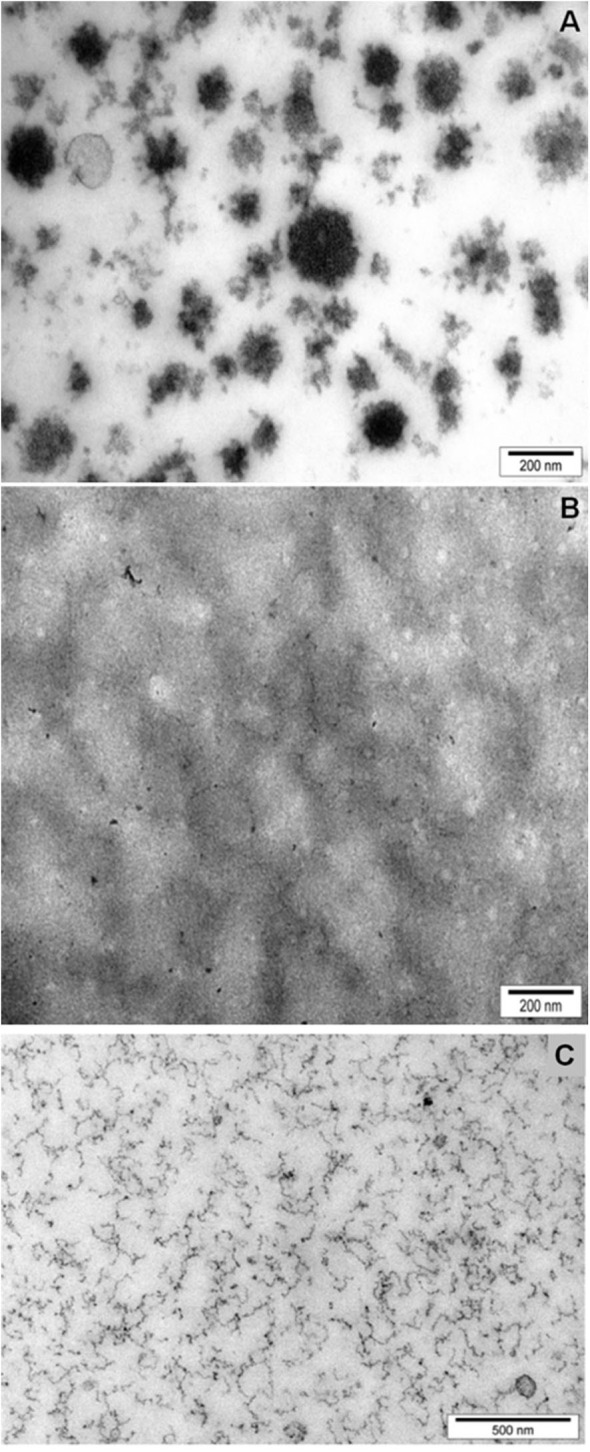
Structure of casein micelles, as revealed by Transmission Electron Microscopy, in skimmed milk **(A)**; calcium-depleted milk **(B)**; and iron (15 mM) added to calcium-depleted milk **(C)**. Reproduced with permission from Mittal et al. (2015), copyright (2015), Elservier Inc.

Mittal et al. (2015) used a novel approach to incorporate iron into natural casein micelles. As iron and calcium bind to similar sites on the casein micelles, a proportion of the calcium was removed from the casein micelles in milk by ion exchange, and then iron was added to bind to the phosphoserine residues of caseins, where calcium is normally bound. The removal of calcium from the casein micelles affected their physicochemical properties; their integrity was retained at up to ~20% calcium depletion, but substantial disintegration occurred at calcium depletion levels of >50%. In the ~20%-calcium-depleted micelles, the majority (>90%) of the added iron bound to caseins within the micelles. In 70%-calcium-depleted milk, most of the iron was bound into dissociated casein particles that remained non-sedimentable upon ultracentifugation and, surprisingly, no protein aggregation was observed. Transmission electron microscopy showed that the binding of iron with dissociated casein particles resulted in the formation of stable, small nanofibrous structures (Figure 2). This discovery formed the basis of a patented technology (Mittal et al., 2017), which has now been commercialized.

### Milk Fat Globules

Milk fat globules are natural structures that are found in milk. They transport the fatty acids, lipid-soluble components (vitamins A, D, E, and K, carotenoids, etc.), and polar lipids that are essential to the early-life development of the neonate. In particular, some specific components of lipids (e.g., sphingolipids) in the fat globules of human milk are critical for neurocognitive development (Schipper et al., 2020); they also play a key role in the gut health and the immunity of infants (Jackson and Nazar, 2006; Guaraldi and Salvatori, 2012). Milk fat globules are large and polydisperse droplets; for example, they can range in diameter from 0.1 to 15 μm in bovine and human milks (Michalski et al., 2005; Singh and Gallier, 2017). The average fat globule diameter in sheep and goat milks has been shown to be smaller than that in bovine milk (bovine, 4.55 μm; sheep, 3.30 μm; goat, 3.49 μm) (Park et al., 2007). However, several other factors affect the size of the milk fat globule, such as stage of lactation and season, among others (Walstra, 1995; Couvreur et al., 2006; Martini et al., 2016).

The core of the fat globule is composed mainly of triacylglycerols (TAG), which account for 98% of the lipid fraction of milk. Milk fat globules are surrounded by a complex interfacial membrane, known as the milk fat globule membrane (MFGM). The assembly of the MFGM occurs in the secretory cells of the mammary glands. The process of the secretion of milk fat globules has been previously reviewed in detail by Heid and Keenan (2005). In brief, milk TAG are produced in the endoplasmic reticulum and are released as microlipid globules that are coated by a single layer of proteins and polar lipids (Masedunskas et al., 2017; Mather et al., 2019). These droplets then move to the apical part of the cell, where some fuse together, resulting in fat globules of different sizes (Masedunskas et al., 2017; Mather et al., 2019). Interactions between the single layer of fat globules and the bilayer of the plasma membrane occur in this part of the cell, resulting in a multilamellar membrane.

It is well-known that common milk processing treatments damage the structure and composition of MFGM and affect the stability of the fat globules (Singh, 2006). The MFGM proteins tend to denature and associate with whey proteins via disulfide bonding during pasteurization and ultra-high-temperature treatment (Corredig and Dalgleish, 1998; Ye et al., 2004), resulting in significant changes in the composition of the MFGM. Cooling of milk also results in the release of MFGM material, including about 20% of the phospholipids, into the milk serum phase (Walstra et al., 2006). Freezing of milk or cream induces crystallization of the TAG and therefore rupture of the MFGM and destabilization of the fat globules upon thawing. Homogenization reduces the size of the fat globules, resulting in a significantly increased surface area. When the MFGM is ruptured, and because of the lack of sufficient MFGM material, the homogenized globules are covered by additional skim milk proteins (Walstra, 1995).

#### Structural Features and Properties

**Figure 3:**
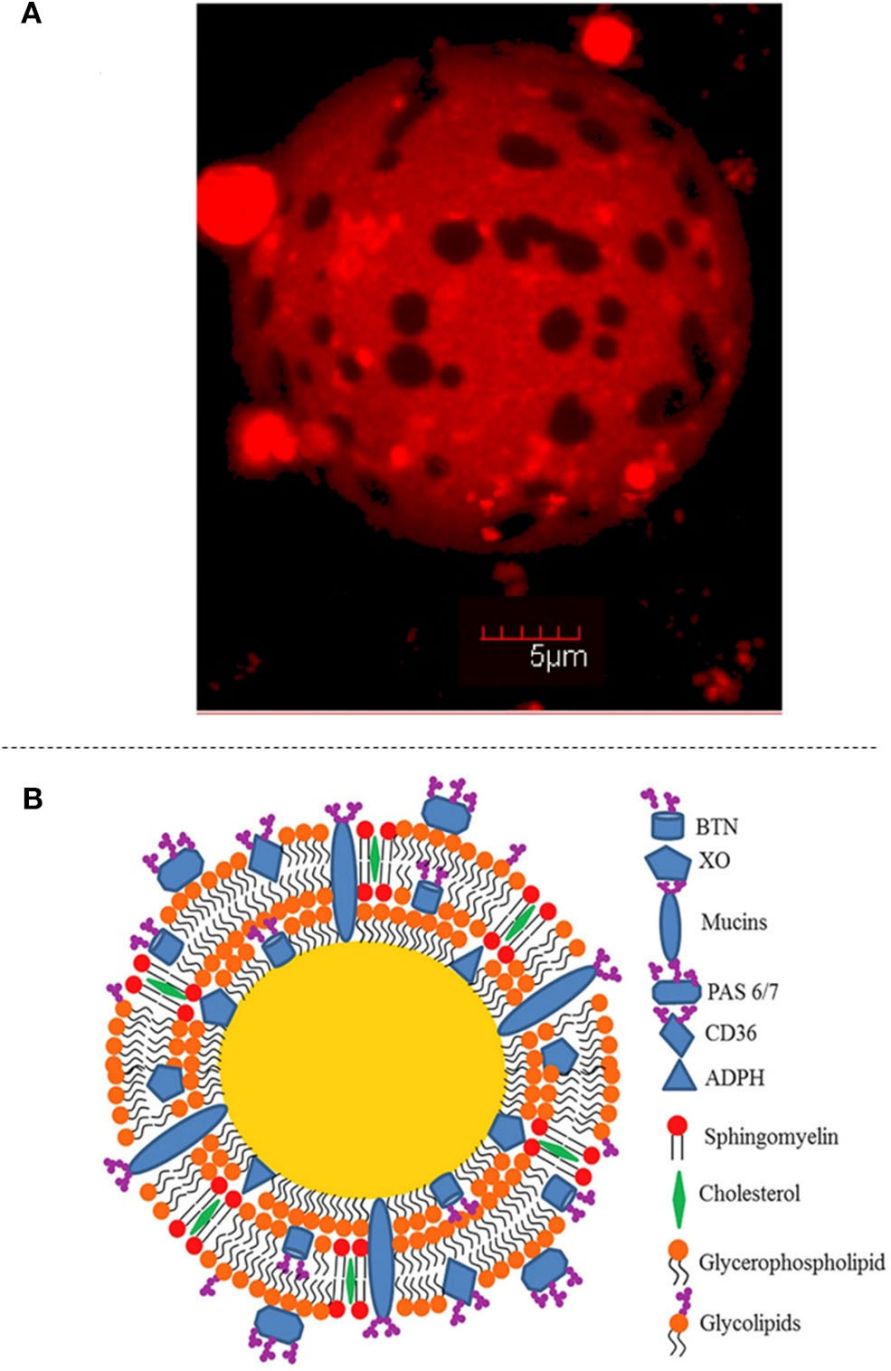
Structure of the milk fat globule. **(A)** Confocal Laser Scanning Microscopy image showing the surface of the milk fat globule, including separated domains. Reprinted with permission from Gallier et al. (2010), copyright (2010), American Chemical Society. **(B)** Schematic representation of the structure of the milk fat globule, highlighting the composition of the membrane. Reproduced with permission from Singh and Gallier (2017), copyright (2017), Elsevier Inc.

Although the MFGM represents only ~2–6% of the fat globule mass, its structure and composition deserve attention. The MFGM is relatively thin, about 8–10 nm thick, and is assembled in a multilayered fashion. Similar to other biological membranes, it is composed of a phospholipid bilayer, with embedded proteins and polar lipids, which is the outer membrane. In addition, there is a third inner monolayer of phospholipids, which is in contact with the triacylglycerol-rich core of the fat globule. A schematic representation of the structure of the MFGM is shown in Figure 3B.

The MFGM consists mostly of membrane-specific proteins, phospholipids, glycoproteins, cholesterol, enzymes, and other minor compounds. The most abundant polar lipids are phosphatidylcholine, phosphatidylethanolamine, and sphingomyelin, whereas phosphatidylserine and phosphatidylinositol are present in minor proportions. Cholesterol has also been described as a major component of the MFGM, and accounts for about 30% of the lipid fraction of the membrane (Yao et al., 2016; Et-Thakafy et al., 2017). The phospholipids are segregated between liquid-ordered domains (particularly rich in sphingomyelin and cholesterol) and liquid-disordered phases (Gallier et al., 2010; Lopez, 2011) (Figure 3A). Proteins represent around 25–60% of the total mass of the membrane; around 100 proteins have been identified using proteomic techniques (Reinhardt and Lippolis, 2006). Xanthine oxidase, butyrophilin, adipophilin, and periodic acid Schiff (PAS) 6/7 represent the major fractions of the proteins that are associated with the MFGM. Polymeric immunoglobulin receptor protein, apolipoprotein E, apolipoprotein A1, 71 kDa heat-shock cognate protein, clusterin, lactoperoxidase, immunoglobulin heavy chain, peptidylprolyl isomerase A, actin, fatty-acid-binding protein, and mucin have been identified as minor fractions (Fong et al., 2007).

From a colloid science viewpoint, the MFGM acts as an emulsifier because the presence of phospholipids and proteins imparts an amphiphilic character. In raw milk, the MFGM prevents the coalescence and flocculation of the fat globules; it also protects TAG located in the core of the globule from oxidative reactions (Lopez, 2005). Another interesting feature is that, during gastrointestinal digestion, the fat globules show some resistance to enzymatic hydrolysis, possibly owing to the presence of the MFGM (Gallier et al., 2012).

It is the structure, composition, and functionality of the MFGM that makes fat globules so interesting for the food industry. This nature-assembled structure has been widely explored as an ingredient for food applications. For instance, an understanding of its composition, structure, and nutritional role has been crucial for the development of infant formulae that closely match human breast milk (Gallier et al., 2015) and for an improvement in the quality of dairy products (Michalski et al., 2004; Romeih et al., 2012; Gauvin et al., 2018). Moreover, the milk fat globule components may also serve as bio-based materials for designing delivery vehicles for bioactive compounds and may enhance their incorporation into foods and their absorption after gastrointestinal digestion.

#### Extraction of MFGM

MFGM material, known for its efficient emulsifying properties, is commonly isolated from byproducts from the dairy industry; in particular, buttermilk and butter serum contain significant proportions of MFGM. Extensive studies on the isolation and purification of the MFGM from milk fat globules have been carried out (Singh, 2006; Dewettinck et al., 2008; Holzmüller and Kulozik, 2016). Several factors that affect the composition and functional properties of MFGM isolates, such as the method of separation, the type of raw material, and the pretreatment of the cream or buttermilk, have been identified. Traditionally, the method of isolation of MFGM from milk at laboratory scale involves the separation of the fat globules by centrifugation, washing with buffer to decrease losses of the MFGM components (milk salts buffer or sucrose solution), disruption of the washed fat globules to release membrane material, and separation of the released material by high-speed centrifugation (90,000–100,000 × *g* for 60 min) or precipitation with acids and salts (e.g., ammonium sulfate) (Singh, 2006). The buffer, the dispersion method, the number of washings, and the type of extraction employed can contribute to the loss of membrane components to various degrees. Differences in the yield and composition of the membrane material are commonly reported. At present, the dairy industry utilizes microfiltration, ultrafiltration, and diafiltration technology for the extraction of MFGM from byproducts. These membrane technologies enable the selective separation of proteins (casein, whey proteins), lactose, and minerals in order to achieve a more pure fraction of MFGM from the byproducts. Spray drying is often applied to MFGM isolate to extend its shelf life (Lopez et al., 2019).

MFGM can be further fractionated into milk phospholipid fractions that are useful for specific applications (e.g., the fabrication of liposomes). Microfiltration coupled with supercritical fluid extraction has been investigated as a method for obtaining a purified fraction of phospholipids (Spence et al., 2009). Microfiltration is utilized for the initial separation of the lipid phase from the proteins in buttermilk, and supercritical fluid extraction utilizing carbon dioxide is used to selectively extract lipid components (such as phospholipids) from a complex mixture. For the microfiltration process, ceramic membranes of 0.45 μm pore size are chosen to lower the transmission of lipids and to decrease fouling of the membrane. Buttermilk passes through the membrane at low temperatures (8–10°C) and transmembrane pressures of 80–95 kPa; this allows a more efficient retention of MFGM lipids. The lipid-rich retentate is dehydrated using the spray drying process. Then, fractions are treated by a supercritical fluid process under certain conditions. Parameters such as pressure, temperature, flow rate, total vessel flushes, and carbon dioxide used need to be optimized to obtain effective separation of the phospholipids from buttermilk powder (Spence et al., 2009). MFGM fractions with different proportions of phospholipids and membrane proteins are now being produced commercially for use in infant formulae and high-value nutritional products.

#### Milk Fat Globules and MFGM Material as Carriers of Bioactive Compounds

The use of intact, natural fat globules to encapsulate and transport exogenous hydrophobic bioactive compounds has not been studied in any detail. Recently, Alshehab et al. (2019) isolated milk fat globules from raw milk using centrifugation (3,000 × *g* for 5 min); an ethanol solution of vitamin D_3_ was incubated with 20% (w/v) cream in water. Entrapment of the vitamin D_3_ occurred via simple diffusion, because the polarity of MFGM changes in the presence of ethanol. A partial opening of the membrane enabled the small hydrophobic molecule to move from the ethanol solution to the core of the fat globule. Following this study, Alshehab and Nitin (2019) showed the efficient encapsulation of curcumin in intact milk fat globules. Confocal laser scanning microscopy analysis revealed small changes in the structure of the curcumin-loaded fat globules during the gastric phase of digestion, followed by major changes in the intestinal phase. Consequently, the release of curcumin was limited in the gastric phase (~27%) and a substantial increase was observed in the intestinal phase (>80%). The use of intact milk fat globules represents an alternative to conventional encapsulation systems (simple, rapid, and low-energy technology). The unique properties of the MFGM may present advantages in improving the delivery of bioactive compounds; however, the structure of the milk fat globule can be easily altered by processing, as discussed above, affecting its specific functions. In view of these challenges, more research is needed on extraction methods that lead to intact fat globules with little membrane disruption and on preservation methods for their utilization at a commercial scale.

Over the past 15 years, studies have shown the potential of the MFGM for the encapsulation of exogeneous bioactive compounds. Oil-in-water emulsions containing model compounds have been successfully formed from MFGM material, in which the hydrophobic molecule is incorporated into the core of the oil droplets. He and Ye (2019) encapsulated β-carotene in a soybean/MFGM material oil-in-water emulsion. Although the efficiency of MFGM as an emulsifier was proven, i.e., a decrease in droplet size as a function of the concentration of the MFGM material, there was a significant destabilization of the emulsion during simulated gastric and intestinal digestion. This led to an early release of free fatty acids and β-carotene in the small intestinal phase.

The application of MFGM phospholipids to make liposomes was first reported by Thompson and Singh (2006). These liposomal structures were successfully used to entrap small hydrophobic (β-carotene) and hydrophilic (potassium chromate) molecules (Thompson et al., 2009). Because liposomes are vesicles consisting of one or more phospholipid bilayers, the structure presents both hydrophilic and hydrophobic domains. Therefore, whereas β-carotene was entrapped within the phospholipid bilayer via hydrophobic interactions, potassium chromate was encapsulated inside the vesicle compartment formed by the polar heads of the phospholipids. The composition of the liposomes formed from MFGM phospholipids was quite different from that of liposomes obtained from egg- or soy-derived phospholipids. Egg-derived phospholipids are composed mainly of phosphatidylcholine and soy-derived phospholipids contain phosphatidylcholine, phosphatidylethanolamine, and phosphatidylinositol; in contrast, MFGM-derived phospholipids are a mixture of phosphatidylcholine, phosphatidylethanolamine, sphingomyelin, and phosphatidylserine (Burling and Graverholt, 2008). This unique composition has been linked to properties that are inherent to MFGM liposomes, such as higher phase transition temperatures, lower membrane permeability, a thicker membrane, and higher physical stability to changes in pH and ionic strength, compared with liposomes prepared with soy-derived phospholipids (Thompson et al., 2006a,b).

Vitamin C, tea polyphenols, lactoferrin, and curcumin have all been successfully entrapped in liposomes of MFGM-derived phospholipids (Farhang et al., 2012; Gulseren and Corredig, 2013; Liu et al., 2013; Jin et al., 2016). Liposomes made from MFGM-derived phospholipids have a greater encapsulation efficiency, have a greater electrical charge, and are relatively smaller than those from lecithin phospholipids. *In vitro* release profiles have shown a slower release of the encapsulated bioactive compound, compared with lecithin phospholipids. This evidence reveals the potential of MFGM-derived phospholipids for the encapsulation of hydrophobic and hydrophilic bioactive molecules. Recently, the glycoprotein fraction in the MFGM has been suggested to be a material with potential for the encapsulation of lactic acid bacteria. The evidence around strong interactions between glycoproteins such as MUC1 and a mucin-binding factor on the surface of the bacteria suggests this material for the enhanced encapsulation of probiotics. In addition, the health benefits brought by membrane-specific proteins may also be an advantage (Guerin et al., 2019).

## Nature-Assembled Structures in Plants

Plants exhibit hierarchical structures assembled by nature, from molecular to macro scale, that are designed in some ways for numerous reasons. It is generally accepted that edible plants structures are divided into two categories: (1) fleshly structures, and (2) encapsulated embryos. While the first category are hierarchical composites of hydrated cells bound by cell walls and middle lamella (e.g., tubers, fruits, vegetables), the second category refers to assemblies of dispersed starch, proteins and lipids into discrete pockets (e.g., cereals, legumes, nuts) (Parada and Aguilera, 2007; Do et al., 2018). These pockets are also referred as starch granules, protein bodies and oil bodies, which act as energy reservoirs of the plants. For the purpose of this review, we will focus the discussion on the oil bodies or olesomes as potential delivery vehicles of bioactive substances.

### Oleosomes or Oil Bodies

Plants of several different species store lipids in discrete organelles called oil bodies or oleosomes (Huang, 1992). These oil-bearing spherical organelles have been found in the cells of different plant tissues, including pollens, flowers, stems, seeds, and fruits (Huang, 1996). The oleosomes from seeds and fruits have been studied extensively because of the potential commercial and nutritional value of the oils from these tissues. The primary function of oleosomes in seeds is to serve as energy reserves that could be used by the plant during seed germination (Huang, 1992). Oleosomes have been of research interest for decades because of their potential to be used as bio-origin delivery systems. As well as being plant oils containing unsaturated fatty acids, oleosomes are naturally emulsified delivery systems for a number of bioactive molecules including vitamin E, carotenoids, phytosterols, and others.

Traditionally, oleosomes can be extracted by grinding presoaked seeds with an aqueous extraction buffer, resulting in an aqueous suspension of co-extracted proteins, oleosomes, and fibrous material. The oleosomes in this suspension can be easily separated by centrifugation and can be used as such after a few washes with plain water or buffer to obtain a “crude extract” of oleosomes. Generally, more extensive washing steps with or without the use of chaotrophic agents and/or organic solvents are used to further purify the oil bodies. Such oleosomes that have undergone one or more purification steps are referred to as “washed oleosomes.” The various methods used for the extraction and purification of oleosomes have recently been reviewed by Nikiforidis et al. (2014). Recently, Ntone et al. (2020) proposed that co-extraction of oleosomes and proteins allowed more efficient extraction of the proteins from the oil seeds.

#### Structural Features of Oleosomes

**Figure 4:**
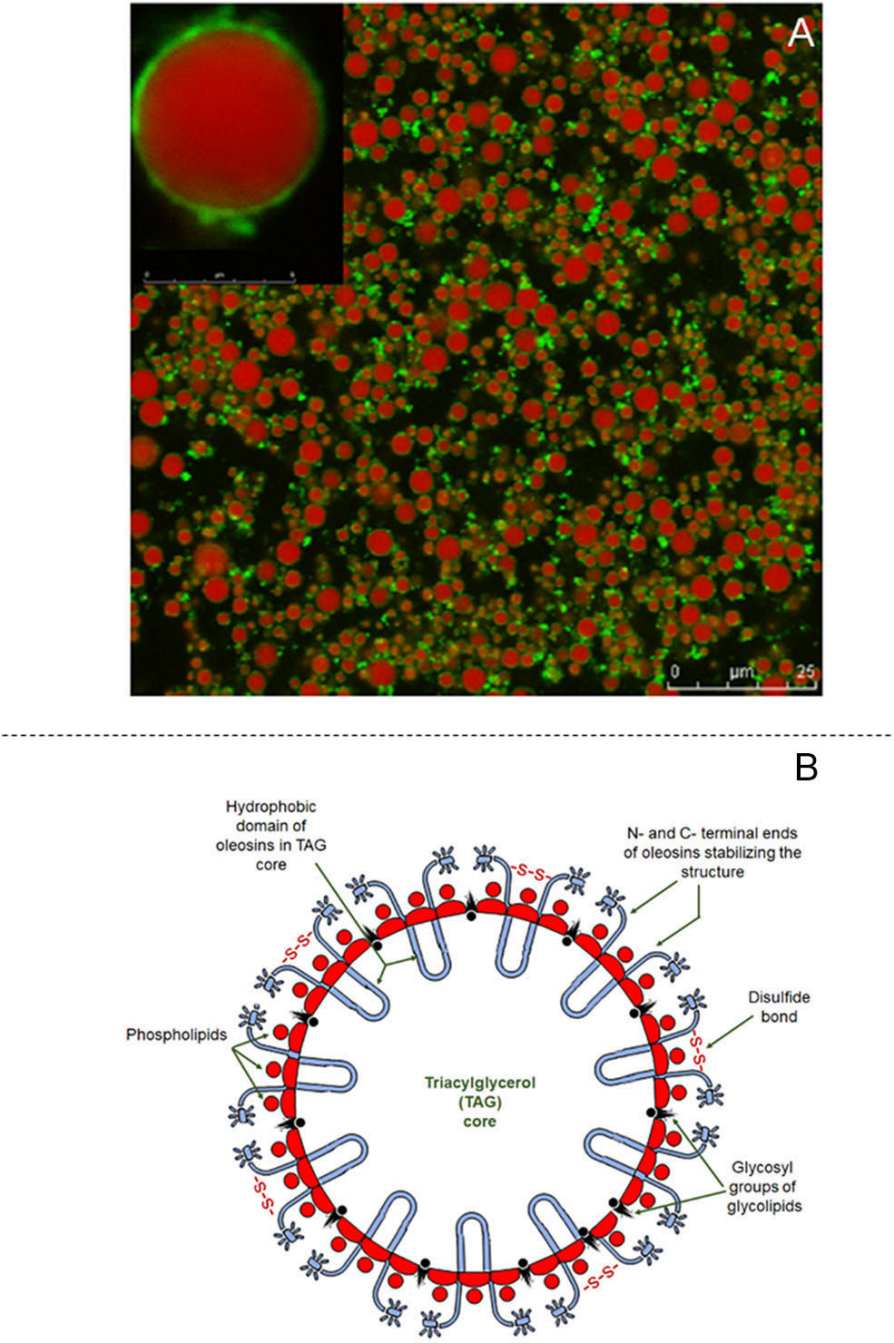
Structural features of oleosomes. **(A)** Confocal laser scanning images of almond oleosomes showing triglycerides core stained in red by Nile Red and oleosins stained in green by Fast Green FCF, reproduced with permission from Gallier and Singh (2012), copyright (2012) Elsevier Inc; and **(B)** Schematic showing structure of coconut oleosome, adapted from Dave et al. (2019), copyright (2019), Elsevier Inc.

The structure of oleosomes shows a high degree of resemblance to that of the milk fat globule of mammalian milk. In aqueous dispersions, oleosomes appear to be spherical and vary in diameter from 0.5–2.0 μm (Huang, 1996) to 10–20 μm in fruits (Ross et al., 1993; Dave et al., 2019). Their structure consists of a central hydrophobic core containing plant TAG, which constitute up to 94–98% (w/w). The triacylglycerol core is stabilized by an interfacial layer consisting of 0.6–2% (w/w) phospholipids and 0.6–3% (w/w) proteins, called oleosins (Tzen et al., 1993). A schematic representation of the structure of the olesome is shown in Figure 4B, while the different components of almond oleosomes, using CSLM, are shown in Figure 4A. The thickness of this interfacial layer is reported to be ~24 nm (Napier et al., 1996).

The biogenesis of oleosomes is a complex process and is initiated by the synthesis of TAG in the endoplasmic reticulum of the cells of oil-bearing plant tissues. Once they have attained a critical size, the oleosomes, covered by a phospholipid monolayer, are released into the cytosol, where fusion of smaller oleosomes could lead to the formation of larger oleosomes (Murphy, 1993). While the oleosomes are in the cytosol, oleosin proteins are deposited on their surfaces, which limits their further growth, resulting in a mature oleosome (Murphy, 1993; Napier et al., 1996). The ratio of TAG to oleosins governs the properties of oleosomes, including their size distribution (Ting et al., 1996), physicochemical stability (Ross et al., 1993; Dave et al., 2019), and mechanical properties (Yang et al., 2020).

The triacylglycerol core of mature oleosomes is surrounded by a monolayer of phospholipids, in which the polar head groups are aligned toward the aqueous phase whereas the non-polar tail remains in contact with the TAG (Tzen and Huang, 1992). Recent characterization studies have shown that the phospholipids present in oleosomes contain a high proportion of saturated fatty acids (Payne et al., 2014; Dave et al., 2019), which allows stronger anchoring of the interfacial proteins and inhibits their detachment by chaotrophic agents, such as urea (Payne et al., 2014). We recently showed that the phospholipid layer of oleosomes isolated from coconut was unaffected by washing with chaotrophic agents and surfactants, such as Tween 20 (Dave et al., 2019).

The major interfacial proteins of oleosomes are oleosins and these proteins are oriented on either side of the phospholipid layer. The oleosins contain a central hydrophobic domain consisting of ~70 residues; this domain remains inside the triacylglycerol core, forming a hairpin-like structure that strongly anchors the oleosins on to the oleosome surface (Tzen and Huang, 1992). In turn, the N- and C-terminal ends remain outside the phospholipid layer, with the N-terminal end being extended out into the aqueous phase (Zielbauer et al., 2018). The interfacial structure of oleosomes is further reinforced by electrostatic interaction between the positively charged residues on the oleosins and the negatively charged phosphate groups of the phospholipids (Ratnayake and Huang, 1996; White et al., 2008). The oleosins stabilize the oleosomes against coalescence by electrostatic repulsion and steric effects (Frandsen et al., 2001; Maurer et al., 2013). Recent studies by our group probed the interfacial composition of oleosomes using different protocols. Our results showed that the surface proteins of coconut oleosomes were disulfide-linked proteins (Dave et al., 2019), which may contribute to the stability of oleosomes. In addition, localization of α-mannopyranosyl and α-glycopyranosyl on oleosomes has also been routinely detected (Gallier et al., 2014a; Dave et al., 2019). The interactions of these functional groups on the oleosome interface are of potential interest for developing new functionalities.

Some recent studies have questioned the need for extensive purification of crude extracts (unwashed) of oleosomes. These extracts have a significant proportion of residual non-oleosome proteins (Nikiforidis et al., 2016) that are linked to oleosins by noncovalent interactions (Dave et al., 2019). It is still unclear whether these non-oleosome proteins should be considered to be part of oleosins. Nevertheless, the washing of oleosomes using chaotrophic agents dislodges these proteins (Dave et al., 2019) and has been reported to cause a decrease in the stability of the oleosomes by promoting flocculation and coalescence (Nikiforidis and Kiosseoglou, 2009; Payne et al., 2014; Zielbauer et al., 2018). This suggests that the non-oleosome proteins linked to the oleosomes play a key role in stabilizing the oleosomes, perhaps through mechanisms similar to those proposed for oleosins. Thus, oleosome-based delivery systems containing relatively crude extracts are likely to remain more stable. This is also desirable for the development of a scalable process for the extraction of oleosomes, because this eliminates the use of non-food-grade chemicals and the cost of associated equipment.

#### Properties of Oleosomes

The physicochemical properties and the stability of oleosomes are greatly affected by the type of method used for extraction. Freshly extracted oleosomes show a high tendency to creaming at neutral pH and flocculate easily during storage (Nikiforidis et al., 2011; Dave et al., 2019). However, the creamed oleosomes remain stable to coalescence and can be redispersed again easily by mechanical agitation (Nikiforidis and Kiosseoglou, 2009). As oleosome dispersions are essentially oil-in-water emulsions that are stabilized by negatively charged (above pH 7) oleosins, their behavior at different pHs and ionic strengths is expected to be similar to that of protein-stabilized emulsions. Oleosomes remain stable at the extremes of pH because of their high electrostatic charge but have a high tendency to flocculate at the isoelectric point or when the charges are screened (Nikiforidis et al., 2011).

Oleosomes have high stability to fluctuations in temperature; the effects of temperature on oleosomes depend on the plant seed species, pretreatments of the seeds prior to extraction, and the extraction methods. Nikiforidis et al. (2011) noted that the structural integrity of crude extracts of oleosomes remained stable to coalescence after a freeze–thaw cycle (−70–40°C), but that repeated cycles of freezing and thawing destabilized the emulsions. This mechanism has been widely exploited to extract the membrane materials or the oil from oleosomes (Onsaard et al., 2006).

In contrast, several studies have reported the relatively high heat stability of oleosomes. Crude extracts of soy and maize oleosomes showed high stability to coalescence even at temperatures as high as 90°C and for durations from 15 min to 1 h (Nikiforidis et al., 2011; Ding et al., 2020). However, the heat-treated oleosomes had a high degree of aggregation because of protein–protein interactions between non-oleosome proteins that adhered to the oleosomes (Nikiforidis et al., 2011; Chen et al., 2012). More extensive heat treatment at 100°C for 15 min caused extensive coalescence in soy oleosomes, suggesting significant structural changes in the interfacial layer at these temperatures (Ding et al., 2020). Thermal treatment tended to improve the oxidative stability of oleosomes, which was attributed to the denaturation of lipolytic enzymes (Chen et al., 2012; De Chirico et al., 2020). These studies suggest that oleosomes may remain stable to coalescence during commonly employed heat-processing temperatures, but may need to be stabilized against creaming and aggregation for long-term storage.

Apart from their stability to physicochemical stress conditions, oleosomes have a robust structure that is highly stable to mechanical and osmotic stresses. The unique structure of the interfacial membrane is flexible, allowing oleosomes to be compressed (Nikiforidis et al., 2013b; Nikiforidis, 2019), enabling their closer packing in the limited space and irregular geometries inside the oil-bearing cells (Nikiforidis et al., 2013b; Dave et al., 2019). Recently, Yang et al. (2020) noted that oleosomes could recover from compressive strains (ε) as large as 0.3. They hypothesized that oleosomes with a higher oil–protein ratio were more rigid. This implies that the size of oleosomes plays an important role in their mechanical properties, with smaller sized oleosomes (e.g., from seeds) probably being more flexible than larger oleosomes (e.g., those from fruits). The mechanical properties of oleosomes are a real advantage when developing new products and processes with oleosomes as carriers of bioactive compounds.

A key characteristic of oleosomes, which makes them especially valuable as a delivery system, is their high stability against oxidative stresses, compared with surfactant-stabilized emulsions. The rate of formation of peroxides and secondary components during storage was significantly lower in oleosomes than in surfactant-stabilized emulsions (Fisk et al., 2008). The high oxidative stability of oleosomes can be attributed to the protective effect arising from oleosins, which act as a barrier to oxidizing agents such as oxygen and hydroperoxides (Gray et al., 2010). In addition, oleosomes usually contain high amounts of naturally present antioxidants, such as carotenoids, and tocopherols, which have a sacrificial effect (Fisk et al., 2006). In crude extracts consisting of unwashed oleosomes, the phenolic compounds that are co-extracted with the oleosomes may improve the oleosome stability by promoting interactions between oleosins and non-oleosome proteins (Fisk et al., 2006).

#### Oleosomes as Carriers of Bioactive Compounds

Another important feature of oleosomes in the development of a functional delivery system is the high permeability of the interfacial membrane to hydrophobic molecules. To achieve encapsulation, the bioactive molecule of interest, usually a hydrophobic compound dissolved in a solvent, is added to an oleosome dispersion. The hydrophobic compound then diffuses inside the oleosomes because of its high affinity to the hydrophobic TAG in the core. Fisk et al. (2011) reported that oleosomes strongly modulate the headspace concentration of flavor volatiles, implying that they serve as carriers of flavor compounds. Oleosomes have also been used successfully for the encapsulation of carotenoids such as astaxanthin and these oleosomes protected the encapsulated astaxanthin against oxidation (Acevedo et al., 2014). In addition, intact oleosomes have been used extensively for cosmetic formulations such as sunscreens (Gruber, 2017). More detailed work to understand the kinetics of the diffusion of hydrophobic compounds and the feasibility of modulating this diffusion is envisaged in the future. This will make it possible to fabricate oleosomes that are targeted for a specific functionality.

Despite being a promising delivery system, the use of intact oleosomes for encapsulation has been limited perhaps because of the greater flexibility offered by other methods, including the ability to fabricate emulsions of a given size with desirable TAG rather than being limited by specific features of oleosomes. The limitations associated with the use of intact oleosomes have prompted the use of oleosome membrane materials for the fabrication of emulsions, similar to that seen with MFGM (see above). The oleosomes extracted from seeds are destabilized using solvents (Tzen and Huang, 1992; Beisson et al., 2001) or repeated cycles of freeze–thaw treatment (Onsaard et al., 2006). The aqueous phase obtained after the removal of the TAG is concentrated and used for emulsification. For ease of distinction, such emulsions that are made using oleosome membrane material are referred to as “artificial oleosomes.” This approach offers several advantages over the use of natural oleosomes, such as the ability to customize the composition of the TAG, the ease of handling the bioactive compounds, which could be added directly to the oils, and the use of conventional equipment in processing. Table 2 summarizes some examples of bioactive molecules encapsulated in artificial oleosomes. Remarkably, most of these studies reported a relatively high stability of the encapsulated bioactive compounds in the oleosomes, suggesting that artificial oleosomes have similar structural features to natural oleosomes.

**Table 2:** Bioactive compounds encapsulated in artificial oleosomes.

**Bioactive compound**	**Outcome/EE**	**References**
Fish oil	Enhanced stability of fish oils to oxidation as compared to small molecule surfactant- or protein-stabilized emulsions	Wijesundera et al., 2013
Curcuminoids	Enhanced stability of curcuminoids against degradation	Bettini et al., 2013
Curcumin	Enhanced stability, high bioavailability of curcumin (rat model)	Chang et al., 2013
Curcumin	Inhibition of growth in HER2/neu-positive tumor cells (*in vitro*)	Chiang et al., 2019
Cyclosporine A	Enhanced bioavailability of the drug (rat model)	Chen et al., 2005
Model hydrophobic molecules	Targeted delivery of hydrophobic molecules	Chiang et al., 2012
Anti-cancer drug Camptothecin	High stability of the drug, oral administration of the drug encapsulated in oleosomes led to regression of tumor in a rat model	Chiang et al., 2011

Although the fabrication of artificial oleosomes is a promising approach, it still needs further investigation. Most of the experimental studies that have been undertaken on artificial oleosomes use either harsh organic solvents or laborious freeze–thaw cycles to break the oleosomes for the extraction of the oleosome membrane material. In addition, once extracted, it is challenging to maintain the stability of the oleosins because of their high tendency to aggregate in an aqueous environment via hydrophobic interactions, which is attributed to their high hydrophobicity (Nikiforidis et al., 2013a). Recently, Ishii et al. (2017) showed that, when oleosomes were homogenized with oil, the interfacial materials were redistributed in the newly formed emulsion droplets. Although this technique appears to be promising for making artificial oleosomes, there needs to be further work to understand the properties of the emulsion droplets obtained and the mechanisms to obtain a similar structure of the droplets to that of oleosomes.

Thus, from the above discussion it is clear that, oleosomes are valuable resources as bio-origin structures, and have the potential to be used as food ingredients. Oleosomes can be extracted from a range of different sustainable sources using existing GRAS materials and processes; they show high stability of the oil and of the bioactive molecule contained within the oil, and good biocompatibility in food formulations. They have interesting material properties that can be exploited for different applications in food, pharma, and cosmetic product formulations. However, several areas remain unexplored, including the ability to fine-tune their properties for a targeted application, the development of a large-scale extraction process for oleosome membrane materials, the stabilization of oleosomes in complex food systems, their compatibility with other food ingredients, e.g., emulsifiers, the properties of artificial emulsions, the dynamics of the digestion of oleosomes in the human gastrointestinal tract, and the bioavailability of bioactive molecules encapsulated in natural or artificial oleosomes.

## Gastrointestinal Behavior of Nature-Assembled Delivery Systems

Understanding the behavior of both natural and engineered delivery systems during digestion in the gastrointestinal tract is critically important for optimization of the bioaccessibility and bioavailability of nutrients and bioactive compounds. The harsh environment of the gastrointestinal tract is designed by nature to break down food materials into small molecular species that can be easily absorbed through the wall of the gastrointestinal tract. The process of digestion and absorption is complex and occurs through several sequential operations, which include mouth, stomach, small intestine, and large intestine (Boland et al., 2014; Boland, 2016).

The pH of the human stomach varies from about 1–3, because of the presence of hydrochloric acid. Such a strongly acidic environment, combined with mechanical action and shear forces caused by peristaltic movements, alters the structures of ingested food materials. The gastric secretion also contains enzymes, pepsin and gastric lipase, which cause the hydrolysis of proteins and lipids, respectively (Bornhorst and Singh, 2014).

The small intestine is the major region in which nutrients and bioactive molecules are released, digested, and converted to an absorbable form. The intestinal fluid is a neutral pH environment (pH 6–7.5) and contains bile salts, pancreatic enzymes, coenzymes, various salts, phospholipids, yeasts, and various bacteria (Ye et al., 2017). Bile salts play a significant role in both the digestion and the adsorption of lipids because of their high surface activity (Sarkar et al., 2016). Bile salts can adsorb readily at the oil–water interface and facilitate the digestion of lipids by providing accessibility of the lipase/colipase complexes to the bile-coated lipid droplets (Sarkar et al., 2016). In this section, we highlight how some of the nature-assembled structures discussed earlier behave in the gastrointestinal tract.

### Casein Micelles

**Figure 5:**
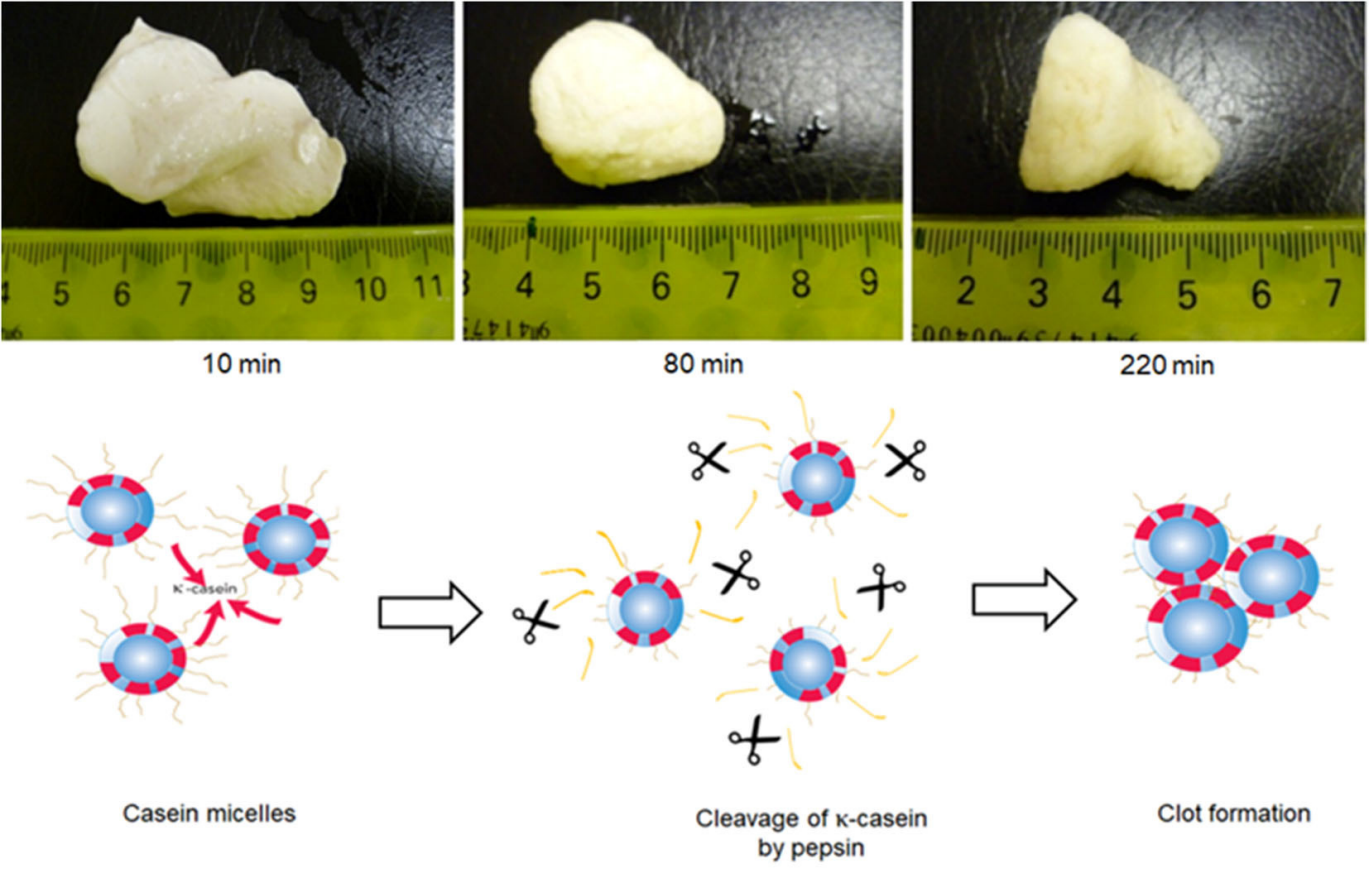
Formation of clots during *in vitro* gastric digestion of skimmed milk in a Human Gastric Simulator. The mechanism of clot formation is also depicted in the figure; κ-casein hairs are clipped from the surface of the casein micelles by the action of pepsin, resulting in the loss of micelle stability and formation of a coagulum Adapted with permissions from Ye et al. (2016a), copyright (2016), Elsevier Inc., and Ye et al. (2020), copyright (2020), Elsevier Inc.

The digestion and the absorption of proteins from milk have been studied extensively, but many of the studies have been performed with purified protein fractions. Relatively less information is available on the disintegration and reorganization of colloidal, assembled structures in milk in the human gastrointestinal tract. As discussed earlier, the casein micelles in milk are prone to destabilization at acidic pH and by the action of specific proteolytic enzymes. Some enzymes, such as chymosin and pepsin, are known to specifically hydrolyze κ-casein into para-κ-casein and a macropeptide. The loss of κ-casein destabilizes the casein micelle, resulting in aggregation or gel/curd formation. These properties are highly significant in the digestion of milk, as both acidic conditions and proteases (e.g., pepsin) exist in the gastric environment (Figure 5).

In recent years, we have reported a series of studies on the digestion of milk and milk protein ingredients (Ye et al., 2016a,b), using the human gastric simulator (Kong and Singh, 2008, 2010); this is a sophisticated *in vitro* model that can closely mimic many relevant factors of gastric physiology, such as mechanical mixing, progressive acidification, and emptying, that might significantly affect the bioaccessibility of nutrients.

The behavior of natural casein micelles under gastric processing conditions can be seen from the digestion studies on skim milk or dispersions of milk protein concentrate powders. When raw skim milk (natural casein micelles) was subjected to gastric digestion in the human gastric simulator, the casein micelles coagulated to form a firm, dense clot within about 10 min of digestion when the pH was >6.2 (Ye et al., 2016a) (Figure 5). At longer gastric digestion times, the clots became smaller, because of the hydrolysis of caseins by pepsin, but their structure became more dense and less permeable because of a further decrease in the pH. As this dense structure reduced the diffusion of pepsin to the interior of the clot, the rate of casein hydrolysis was slower from the interior of the clot than from the surface of the clot. Recently, Wang et al. (2018) showed that skim milk reconstituted from skim milk powder or milk protein concentrate solution (both of which contain intact casein micelles) also formed a cheese-like curd with a close-knit network at an early digestion time and that a long time was required to fully disintegrate this curd structure. At pHs below 5.0, most of the MCP would be expected to migrate from the colloidal phase to the soluble phase and to empty out of the stomach rapidly.

Furthermore, the properties of the curd formed in the stomach can be manipulated by the pretreatment of the milk. For example, skim milk heated at 90°C for 20 min formed a looser, fragmented network-structured clot with numerous larger voids, compared with unheated milk, which formed a firm, dense-structured clot (Ye et al., 2016a). This effect is related to the association of the whey proteins with the casein micelles during heat treatment (Anema and Li, 2003a,b; Donato et al., 2007). Consequently, the hydrolysis of the caseins was much faster in heated milk than in unheated milk, essentially because the open, fragmented structure increased the effective contact area with the simulated gastric fluid, which contained pepsin. Pepsin was thus able to diffuse into and act on the proteins within the clot.

Casein-micelle-based gelled structures (acid gel vs. rennet gel) have been shown to behave differently during digestion, opening up the possibility of using these systems for bioactive compound delivery (Barbé et al., 2014). Using *in vivo* models (mini-pigs), the ingestion of the rennet gel resulted in lower levels of proteins in the duodenum and lower levels of amino acids in the plasma, compared with the ingestion of the acid gel, even though the two gels had the same composition and similar rheological and structural properties. It was suggested that a coagulum with high stiffness was formed after the ingestion of the rennet gel, under the simultaneous action of the stomach acidity and the rennet, leading to a very long retention of the rennet matrix in the stomach.

Clearly, the formation of a clot by casein micelles in the stomach and its gradual slow degradation with digestion time provides a mechanism for the controlled delivery of protein to the small intestine. It would be expected that any bioactive compound encapsulated/bound within the casein micelles would remain entrapped in the curd, and would be released slowly as the protein network is hydrolyzed. Moreover, the structure of the curd can be manipulated by preheat treatments, providing possibilities for developing delivery systems for the controlled release of bioactive molecules. Further research in this area is warranted, because, to date, very few publications on this topic are available.

### Milk Fat Globules

Several aspects of the digestion of milk fat globules have been extensively investigated, whereas others still need further investigation. For example, digestion in the oral cavity has not yet been explored in depth. In a study published by Smoczynski and Staniewski (2014), it was reported that milk fat globules experienced reversible flocculation after mixing with artificial saliva, which was attributed to depletion flocculation. Nonetheless, the extent of these interactions is highly dependent on the food matrix in which the milk fat globules are present. Liquid foods (e.g., milk) will have negligible residence time in the oral cavity, compared with semi-solid and solid foods (e.g., yogurt or cheese); consequently, flocculation may occur to a lesser or a greater extent. Clearly, the behavior of milk fat globules during oral digestion needs to be explored further.

**Figure 6:**
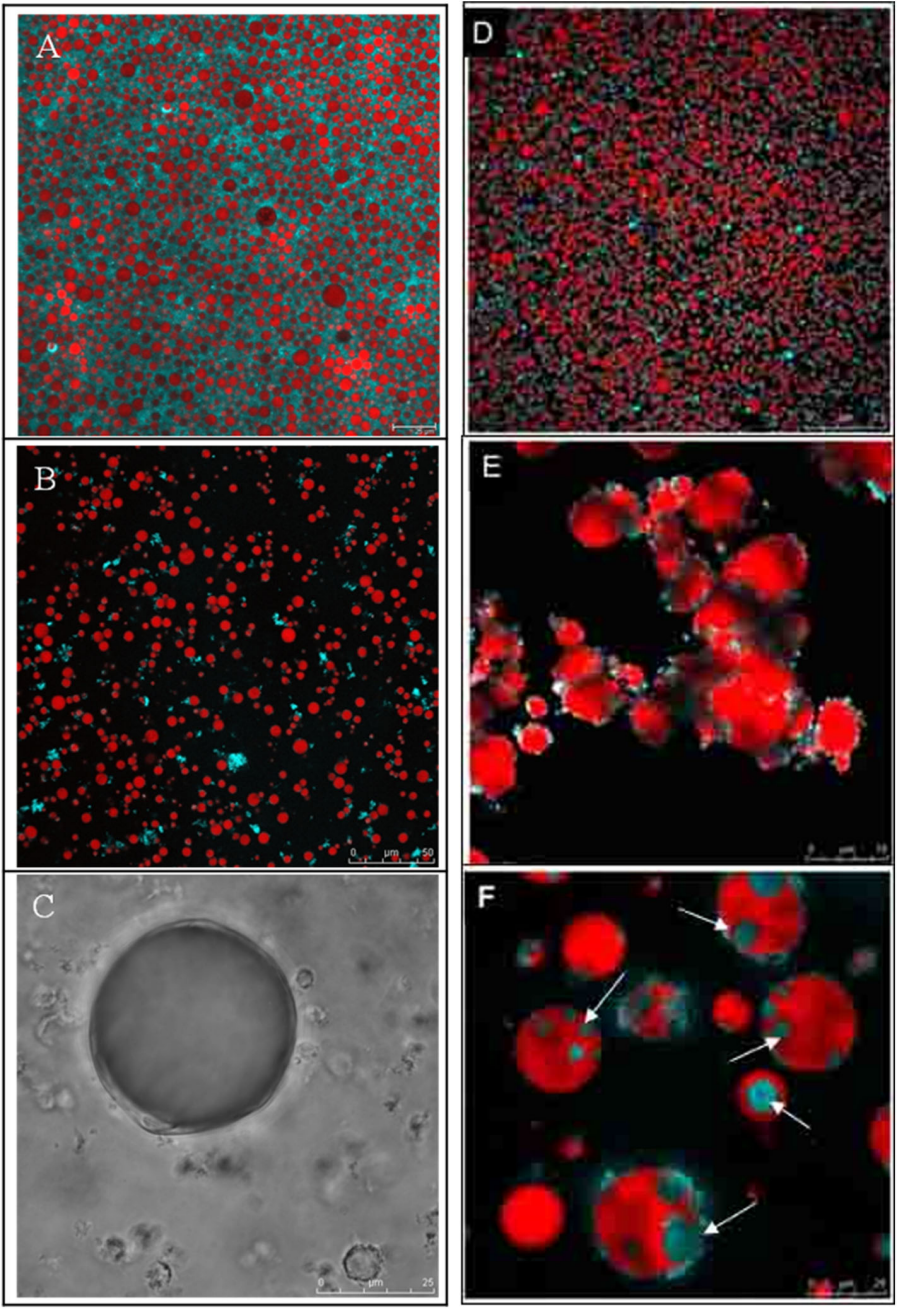
Confocal Laser Scanning Microscopy images of bovine milk fat globules **(A–C)** and walnut oleosome dispersions **(D–F)** in native conditions **(A,B)**, after 60 min of gastric digestion **(B,E)**, and after 60 min of gastric digestion followed by 30 min of intestinal digestion **(C,F)**. The white arrows in **(F)** show spontaneous biological multi-phase emulsions formed as a result of interactions of fatty acids and bile salts. Adapted with permissions from Gallier et al. (2012), copyright (2012), Elsevier Inc. **(A–C)**; and Gallier et al. (2013b), copyright (2013), American Chemical Society **(D–F)**.

Milk fat globules have shown different behaviors in the gastric environment, depending on the level of processing (native state vs. processed state) and the presence of other food components such as milk proteins. Milk fat globules in their native state, i.e., such as those present in raw milk, have been shown to be relatively resistant to gastric digestion (Figures 6A–C). Although gastric pepsin hydrolyzes MFGM proteins, the peptides remain on the surface of the globules, contributing to their stability. Ye et al. (2011), Gallier et al. (2012) demonstrated that the membrane-specific proteins of the fat globules in raw milk were hydrolyzed into various peptides after *in vitro* digestion. However, the rate of proteolysis differed among MFGM proteins. For instance, xanthine oxidase was hydrolyzed much faster than butyrophilin, and PAS 6 and PAS 7 were hydrolyzed faster than butyrophilin, but PAS 7 was more sensitive than PAS 6 (Ye et al., 2011; Gallier et al., 2012). In addition, it has been shown that not all proteins are hydrolyzed by gastric pepsin, especially the glycoproteins MUC1 and PAS 6/7 (Le et al., 2012). Microscopic examination of native milk fat globules during digestion has shown increased flocculation as a function of the digestion time but the absence of coalescence (Ye et al., 2011) (Figures 6A–C). This seems to suggest that native milk fat globules are somewhat resistant to gastric digestion because of the stabilizing effect provided by the peptides, proteins, and phospholipids located at the surface of the globules.

As mentioned earlier, heat treatments and homogenization affect the structure of the MFGM, which has been shown to change the gastric behavior compared with that of native milk fat globules. Gallier et al. (2013a) investigated the *in vivo* gastric digestion of bovine milk fat globules in cream derived from either raw milk or heated milk (63°C for 30 min). Fasted rats were orally gavaged once with one of the cream preparations, and stomach chyme samples were collected from the rats post-euthanasia after 30 min, 2 h, and 3 h post-gavage. The results showed that the surface of the native milk fat globules appeared to be less affected by the hydrolytic action of pepsin than that of the homogenized milk fat globules, which were covered by intact milk proteins and their peptides formed by pepsin digestion.

Our recent work, using the human gastric simulator, has shown that, when the fat globules within liquid milk are digested (Ye et al., 2016b), some of the natural fat globules become embedded in the casein matrix/clots (as discussed above) during the early stages of gastric digestion. With an increase in the digestion time, the clot becomes denser and the fat globules within the matrix tend to aggregate and coalesce. It has also been shown that the globules are not part of the clot matrix, but are trapped within the protein matrix of the clot. Interestingly, the release of the fat globules from the protein matrix is directly related to the extent and rate of protein hydrolysis or the breakdown of the clot matrix structure. The rate of droplet release is dependent on the pretreatment of the whole milk prior to digestion, such as heat treatment and homogenization.

Common processing methods applied in the dairy industry disrupt the native complex and the specific configuration of proteins and phospholipids forming the MFGM, that in turn, affect its functional properties and stability during digestion. For example, the fat globules become coated by caseins in homogenized milk because there is insufficient membrane material to cover the newly formed fat globules created by the homogenization. Therefore, they can rapidly aggregate with caseins at their isoelectric point (pH 4.6), but native fat globules aggregate at a lower rate, as shown in previous studies (Armand et al., 1996; Gallier et al., 2013a). These properties may be an advantage to the delivery of bioactive compounds that undergo chemical degradation under gastric conditions, offering enhanced protection to the encapsulated component.

In the small intestine, most gastric-resistant peptides and proteins are cleaved by pancreatic enzymes (trypsin, chymotrypsin, and other proteases) into amino acids, except some glycoproteins (Le et al., 2012). Phospholipids from the MFGM are also digested at this stage; in particular, hydrolysis of glycerophospholipids occurs in the small intestine, mediated by phospholipase A_2_. Sphingomyelin is hydrolyzed in the large intestine by sphingomyelinase (Kuchta et al., 2012). The TAG are also hydrolyzed by pancreatic lipase, releasing two free fatty acids and one 2-monoacylglycerol. Several other active lipases, such as phospholipase A2, cholesterol esterase, and pancreatic-lipase-related protein, are part of the pancreatic juice and contribute to the overall lipid digestion.

Homogenization and pasteurization have been shown to have a significant impact on the intestinal digestion of milk fat globules. Because the lipolysis process occurs at the interfacial level, the composition and structure of the MFGM and the size of the fat globules affect the rate of lipolysis (Bourlieu and Michalski, 2015; Singh and Gallier, 2017). Homogenization increases the surface area of the milk fat globules, compared with that found when the milk fat globules are unprocessed. Therefore, more interface, on which the lipase can anchor, is available, which results in a faster rate of lipolysis of the homogenized fat globules, compared with that observed for non-homogenized fat globules (Ye et al., 2010; Bourlieu and Michalski, 2015).

Gallier et al. (2013c) examined the upper and lower small intestinal contents of rats gavaged with different cream preparations (untreated, pasteurized, and homogenized). Regardless of the treatment of the cream, liquid–crystalline lamellar structures were observed during digestion. This was related to the accumulation of lipolytic products at the interface of milk fat globules, calcium, and bile salts. It has also been observed that homogenization affects the intestinal digestion of milk fat globules more than does pasteurization, as demonstrated by Tunick et al. (2016).

Another important aspect in the intestinal digestion of native milk fat globules that needs to be highlighted is the effect of different sizes and curvatures of the milk fat globules on the delivery of lipids. The various populations of fat globules that are naturally present in milk (small and large sizes) enable a controlled release of lipids to the intestine for further absorption. These characteristics may be of interest for the delivery of bioactive compounds and may be an advantage over homogenized milk fat globules, which typically have a narrow droplet size distribution.

### Oleosomes

An understanding of the digestion behavior of oleosomes is key to tailoring the functionalities of oleosomes to targeted delivery applications. As the oleosome surface is entirely covered with oleosins, their behavior under gastrointestinal conditions is expected to be similar to that of protein-stabilized emulsions. The process of digestion starts with the mastication of food, which breaks the structure of the food into smaller particles and mixes these particles with saliva, resulting in the formation of a bolus and the equilibration of the bolus with the body temperature (van Aken, 2010). Although the mechanical forces during mastication are expected to expel the oleosomes from the seed matrix, recent studies have shown that these forces are insufficient to disintegrate the seed matrix and hence interfere with the gastrointestinal digestion of oleosomes (Grundy et al., 2015). The effect of plant structures on the digestibility and bioaccessibility of nutrients in cells has been reviewed elsewhere (Ogawa et al., 2018; Holland et al., 2020) and the discussion here is largely restricted to the digestibility of extracted oleosomes. During oral digestion, the oleosomes are expected to remain stable because of the near neutral pH of the oral phase (Singh and Gallier, 2014), the absence of any proteases or lipases in the oral phase, and the relatively short residence times in the mouth.

Several studies have investigated the stability of oleosomes under gastric conditions by mimicking gastric conditions *in vitro*. All of these studies used static *in vitro* digestion models and investigated the structural stability of oleosomes under *in vitro* gastric conditions. However, these studies failed to elucidate the physicochemical changes in oleosomes prior to their digestion in the stomach and this remains an area to be explored in detail. Gastric digestion is a dynamic process, with a gradual change in pH with progressive digestion. Our recent work using the dynamic *in vitro* gastric digestion model on a human gastric simulator showed that the *in vitro* gastric conditions have a significant impact on the structural stability of oleosomes and on the composition of the digesta being emptied out in the duodenum (Wang et al., 2020).

The behavior of oleosomes in a dynamic gastric digestion system can be predicted based on our knowledge of structural features. As digestion progresses, the secretion of acids in the stomach causes a gradual reduction in the pH, which, in turn, further contributes to the decline in the surface charges on the oleosomes until the isoelectric pH (between pH 5 and pH 4) is reached. The net effect of the lack of a stabilizing effect of the oleosins is the significant flocculation of the oleosomes via hydrophobic interactions, causing the oleosomes to form a cream phase in the stomach. In addition, in unstructured systems containing oleosomes, the oleosomes may show a high tendency to creaming because of their larger size. The net effect of creaming is a delayed emptying of the oleosomes from the stomach. Nevertheless, as digestion is a dynamic process and the gastric contents are emptied out of the stomach to the duodenum continuously, some oleosomes may be emptied out before the pH of the stomach becomes sufficiently low to facilitate pepsin action.

Lowering the pH below 4 facilitates the action of pepsin on the oleosomes, which may bring about a destabilization of the oleosomes. At sufficiently low pH (pH <4), the proteolytic action of pepsin on the stabilizing hydrophilic ends of oleosins promotes flocculation and aggregation of the oleosomes (Gallier and Singh, 2012; Gallier et al., 2013b) (Figures 6D–F). As the highly hydrophobic sequence of oleosins remains buried inside the triacylglycerol core, away from the aqueous phase, a high structural stability of the oleosomes in the presence of pepsin may be expected. Indeed, the *in vitro* digestion studies on crude oleosome extracts do report limited hydrolysis of oleosins (Gallier and Singh, 2012; Makkhun et al., 2015). In contrast, several studies have reported the coalescence of oleosomes because of the action of pepsin (White et al., 2009; Gallier et al., 2014b; Makkhun et al., 2015; Wang et al., 2020). These results suggest that the stability of oleosomes under gastric conditions may depend on the non-oleosome proteins, which may provide protection against pepsin hydrolysis. In the absence of this protective effect, the oleosins may be susceptible to pepsin hydrolysis, which may destabilize the oleosomes and result in coalescence.

The major implication of the destabilization of oleosomes under gastric conditions is an increase in the droplet size, contributing to creaming and subsequently to the release of free oil with continued digestion. This enhances the susceptibility of the released TAG to gastric lipase, which has significant residual activity at pH < 4. Pafumi et al. (2002) reported that gastric lipase activity may account for the lipolysis of 10– 40% of TAG. The extent of the release of lipid hydrolysis products from oleosomes remained largely unexplored in the *in vitro* studies above, perhaps because of the unavailability of a suitable lipase with characteristics similar to those of human gastric lipase. Recently, Grundy et al. (2016) reported a greater degree of lipid hydrolysis during the intestinal digestion of almond bits when rabbit gastric lipase was used in the preceding gastric digestion. More detailed *in vitro* gastric digestion studies with rabbit gastric lipase are necessary to understand the hydrolysis of lipids from oleosomes in order to be able to design a successful delivery system.

Once in the duodenum, the mixing of intestinal fluid and bile disperses the oleosome flocs rapidly, which has been attributed to an increase in the zeta-potential (the zeta-potential becomes more negative), brought about by an increase in pH (pH 7) and the replacement of interfacial materials by bile salts (Gallier and Singh, 2012). Gallier and Singh (2012) hypothesized that the hydrophobic domains of oleosins anchored in the triacylglycerol core may contribute to a delay in the onset of lipolysis. The adsorption of bile salts on to oleosomes is expected to facilitate the digestion of lipids by displacing the interfacial oleosins and phospholipids and to facilitate the anchoring of the colipase and the lipase (Wilde and Chu, 2011; Gallier and Singh, 2012; Makkhun et al., 2015). The bile salts then promote lipolysis by rapidly removing the free fatty acids and monoacylglycerols in mixed micelles of bile salt and phospholipids. Gallier et al. (2013b) reported the formation of water-in-oil-in-water emulsions called “spontaneous biological multi-phase emulsions” during the intestinal digestion of a walnut oleosome dispersion. These spontaneous biological multi-phase-emulsions entrapped proteins and were probably formed as a result of interactions between specific polyunsaturated fatty acids and bile salts.

Because the digestion of lipids is an interfacial process, the digestibility of oleosomes and hence the bioaccessibility of bioactive compounds encapsulated inside the hydrophobic core of oleosomes can be suitably modulated by altering the surface properties of the oleosomes. The chemical and enzymatic cross-linking of surface proteins has been proposed as one of the approaches for modulating the digestibility of oleosomes (Beindorff et al., 2007), but such an approach is limited because of one or more regulatory hurdles. In comparison, a more promising approach to modulate digestion processes has been exploiting the surface interactions of oleosins with polysaccharides (Ding et al., 2019).

## Conclusions

Consumers demand foods that can improve their health with minimal impact on the environment. This is reinforcing the importance of utilizing natural materials, biocompatible and energy/cost-effective processes to develop nourishing and functional foods. This review highlighted various versatile and nature-assembled structures, namely casein micelles, milk fat globules and oleosomes, with great potential for the delivery of bioactive food compounds. Casein micelles in milk are remarkably complex structures designed by nature to deliver bioactive compounds to the neonate to ensure proper growth and development. The dissociation-association properties of casein micelles have been explored for binding/encapsulation of bioactive molecules, but the highly porous nature of the casein micelles and the vulnerability of their structure to changes in the environmental conditions restrain their applications as delivery systems. Native casein micelles have also shown considerable advantages, but further understanding of their ability and the mechanisms of the binding of hydrophobic molecules within casein micelles is required. Recent studies indicate the formation of a clot by casein micelles in the stomach; its gradual degradation with digestion time provides a mechanism for the controlled delivery of proteins and bioactive compounds encapsulated/bound within casein micelles. Interesting, heat treatment can be used to alter the structure of the clot and therefore the delivery rates. Further research in this area is warranted to develop casein micelle-based delivery systems that provide controlled release of bioactive molecules, through manipulation of the structure of the clot in the stomach.

Milk also contains a nature-assembled system, the milk fat globule, to deliver energy and lipo-soluble vitamins. The membrane (MFGM) surrounding these fat globules has received remarkable attention because the complexity of its structure provides unique emulsifying and biological functions. Native/intact milk fat globules are resistant to gastric digestion, a feature mainly attributed to the structure of the MFGM. The unique properties of the milk fat globules may present advantages in improving the delivery of hydrophobic bioactive compounds. However, the structure of the milk fat globule can be easily altered by processing, affecting its specific properties and functions. Therefore, more research is needed on the extraction methods that lead to intact fat globules with little membrane disruption and on the preservation methods for their utilization at a commercial scale. Most recent studies have shown the potential of intact milk fat globules and other colloidal structures prepared with MFGM on the encapsulation of hydrophobic and hydrophilic compounds, indicating the great potential of milk fat globules as a functional food ingredient.

Another example of the nature-assembled structures is the oleosomes mainly found in the oil seeds. A key characteristic of oleosomes, which makes them especially valuable as a delivery system, is their high stability against oxidative stresses, compared with surfactant-stabilized emulsions. This stability is provided by the oleosin proteins located at the interface of the oleosomes, which act as a barrier to oxidizing agents, such as oxygen and hydroperoxides. It is clear that oleosomes are valuable resources and have the potential to be used as food ingredients. Oleosomes can be extracted from a range of different sustainable sources using existing GRAS materials and processes. However, several areas remain unexplored, including the development of a large-scale extraction process for oleosomes, the stabilization of oleosomes in complex food systems, their compatibility with other food ingredients, the dynamics of the digestion of oleosomes in the human gastrointestinal tract, and the bioavailability of bioactive molecules encapsulated in natural or artificial oleosomes.

It must be remembered that the bioactive delivery system is a just a vehicle and it needs a food matrix or a dietary supplementary format for human consumption. There is a large amount of information already published on extraction and characterization of bioactive compounds from food sources. Also many food materials have been exploited to create “engineered” delivery systems to protect these compounds from harsh processing and storage conditions. However, a few studies have addressed the important issues related to the incorporation of delivery systems into real functional foods. Some of the nature-assembled systems discussed in this review may have the potential to improve solubility, enhance bioavailability, improve controlled release and enable greater precision targeting of entrapped compounds. Much of the current work on bioavailability is focused on cell-line models or rat assays. In future, bioavailability studies will need to be carried out in human clinical trials. The consideration of the regulatory status of the bioactive compounds and the delivery format is also critically important in the development of functional foods.

Longer term opportunities may be in developing functional foods designed for the individual (personalized health). The role of nutritional science may be to build a body of knowledge and ultimately functional foods that enable humans to achieve optimal health status within the lifestyle that each aspires to.

## Author Contributions

AA-F: conceptualization, funding acquisition, writing—original draft, writing—review and editing—final draft, and writing. AD: conceptualization, writing—original draft, and writing—review. HS: conceptualization, funding acquisition, writing—original draft, and writing—review. All authors contributed to the article and approved the submitted version.

## Conflict of Interest

The authors declare that the research was conducted in the absence of any commercial or financial relationships that could be construed as a potential conflict of interest.
